# Skin Electrical Resistance Measurement of Oxygen-Containing Terpenes as Penetration Enhancers: Role of Stratum Corneum Lipids

**DOI:** 10.3390/molecules24030523

**Published:** 2019-01-31

**Authors:** Xue-min Zhu, Yu Li, Fei Xu, Wei Gu, Guo-jun Yan, Jie Dong, Jun Chen

**Affiliations:** 1 Pharmaceutical Research Laboratory, School of Pharmacy, Nanjing University of Chinese Medicine, Nanjing 210023, China; zhuxmyj@163.com (X.-m.Z.); 13912994230@163.com (Y.L.); 300091@njucm.edu.cn (F.X.); guwei@njucm.edu.cn (W.G.); joun.yan@163.com (G.-j.Y.); 13915941917@139.com (J.D.); 2 Jiangsu Provincial Key Laboratory of Chinese Medicine Processing, Nanjing University of Chinese Medicine, Nanjing 210023, China; 3 Jiangsu Key Laboratory for Pharmacology and Safety Evaluation of Chinese Materia Medica, School of Pharmacy, Nanjing University of Chinese Medicine, Nanjing 210023, China; 4 Jiangsu Collaborative Innovation Center of Chinese Medicinal Resources Industrialization, Nanjing University of Chinese Medicine, Nanjing 210023, China

**Keywords:** oxygen-containing terpenes, penetration enhancers, stratum corneum lipids, skin electrical resistance

## Abstract

The measurement of skin electrical resistance (SER) has drawn a great deal of attention for the rapid screening of transdermal penetration enhancers (PEs). However, the mechanisms underlying the SER measurement are still unclear. This study was to investigate the effects and mechanisms of seven oxygen-containing terpenes on the SER kinetics. Stratum corneum (SC) lipids were proved to play a key role in SER measurement. Then, the factors affecting the SER measurement were optimized. By the determination of SER kinetics, cyclic terpenes (1,8-cineole, terpinen-4-ol, menthol and α-terpineol) were demonstrated to possess higher enhancement ratio (ER) values compared with linear terpenes (linalool, geraniol and citral). For the first time, the linear correlation was found between ER of terpenes and the interaction energy of terpene–ceramide complexes revealed by molecular simulation. The attenuated total reflection-Fourier transform infrared spectroscopy (ATR-FTIR) analysis revealed that the effect of cyclic terpenes on SC lipid arrangement was obviously stronger than that of linear terpenes. In addition, by evaluating HaCaT skin cell viability, little difference was found between the toxicities of cyclic and linear terpenes. In conclusion, measurement of SER could be a feasible approach for the efficient evaluation of the PEs that mainly act on SC lipids.

## 1. Introduction

Transdermal drug delivery (TDD) offers several advantages over other routes of drug administration, such as avoiding gut and hepatic first-pass metabolism, improving patient compliance, decreasing administration frequency and reducing side effects. For some compounds, TDD is an effective and preferred route of administration [1]. However, application of TDD is limited primarily by low skin permeability. The skin acts as an effective barrier to protect the organism against undesirable effects of the environment. The main permeation barrier resides in the outermost layer of the skin, stratum corneum (SC), a composite of proteins and lipids in which protein-rich corneocytes are surrounded by lipid bilayers. Ordered structure and low permeability of these lipid bilayers are responsible for the barrier function of the SC [2,3]. To penetrate through the SC, drugs must navigate through the tortuous lipid pathways surrounding the keratin-rich cells, or repeatedly partition between the aqueous, keratin-rich phase into the lipid phase. Unfortunately, most topically administered drugs are unable to penetrate the SC. Many strategies have been developed to improve percutaneous absorption. Among them, penetration enhancers (PEs) have been extensively investigated in the past couple of decades [4].

Efforts have been directed at identifying safe and effective PEs from both natural products and synthetic chemicals. Due to their high penetration enhancement effect and low skin irritation, terpenes of natural origin, especially oxygen-containing terpenes [5], have been receiving more and more attention in pharmaceutical and cosmetic formulations [5,6]. In addition, quite a few oxygen-containing terpenes are included in the list of Generally Recognized As Safe (GRAS) agents issued by the Food and Drug Administration (FDA).

The structure of the SC is simply regarded as a “brick and mortar” model, in which protein-rich corneocytes are the bricks and intercellular lipids are the mortar. The barrier function of SC is mostly attributed to the existence of intercellular lipid matrix [7]. It has been proved that the measurement of electrical resistance across skin membranes provides a more robust and practical measurement of skin barrier integrity compared to the measurement of flux, a standard and conventional means of evaluating skin integrity [8].

The classical permeation experimental apparatus using Franz diffusion cells provides a reliable in vitro technique for screening of PEs for TDD formulations. However, it is labor-intensive and time-consuming. Since PEs usually enhance skin permeability by altering the SC structure, such as affecting the skin integrity, measurement of the changes in skin electrical resistance (SER) has been widely used as an efficient technique to screen effective PEs [9,10,11,12]. However, until now, the mechanisms underlying the SER measurement are still not fully revealed.

Therefore, in this study, we evaluated the factors affecting SER measurement and explored the SER kinetics of seven oxygen-containing terpenes, including cyclic and linear terpenes (Table 1). The SER is determined using a Millicell–ERS device which has been successfully applied for the assessment of skin barrier function as previously described [13]. Furthermore, the mechanisms underlying SER kinetics are intensively investigated in this study.

## 2. Results

### 2.1. Effect of SC and SC Lipids on the Skin Electrical Resistance

To determine whether SC lipids were the key factors affecting skin barrier function, the SER values were measured before and after the removal of SC lipids by organic solvents. It was found that the SER after extraction of SC lipids was reduced to 10.16% compared with the untreated skin. Furthermore, after removal of SC, the SER value reduced to only 1.25%. It was proved that the SER was mainly mediated by SC, especially SC lipids.

### 2.2. Factors Affecting Skin Electrical Resistance Kinetics

Both 1,8-cineole and citral are the main active components of essential oils (EOs) [14]. Moreover, they are usually used as PEs [15,16]. Therefore, factors affecting the SER kinetics of 1,8-cineole and citral were investigated.

#### 2.2.1. Effect of the Temperature on SER Kinetic Measurement

**Figure 1:**
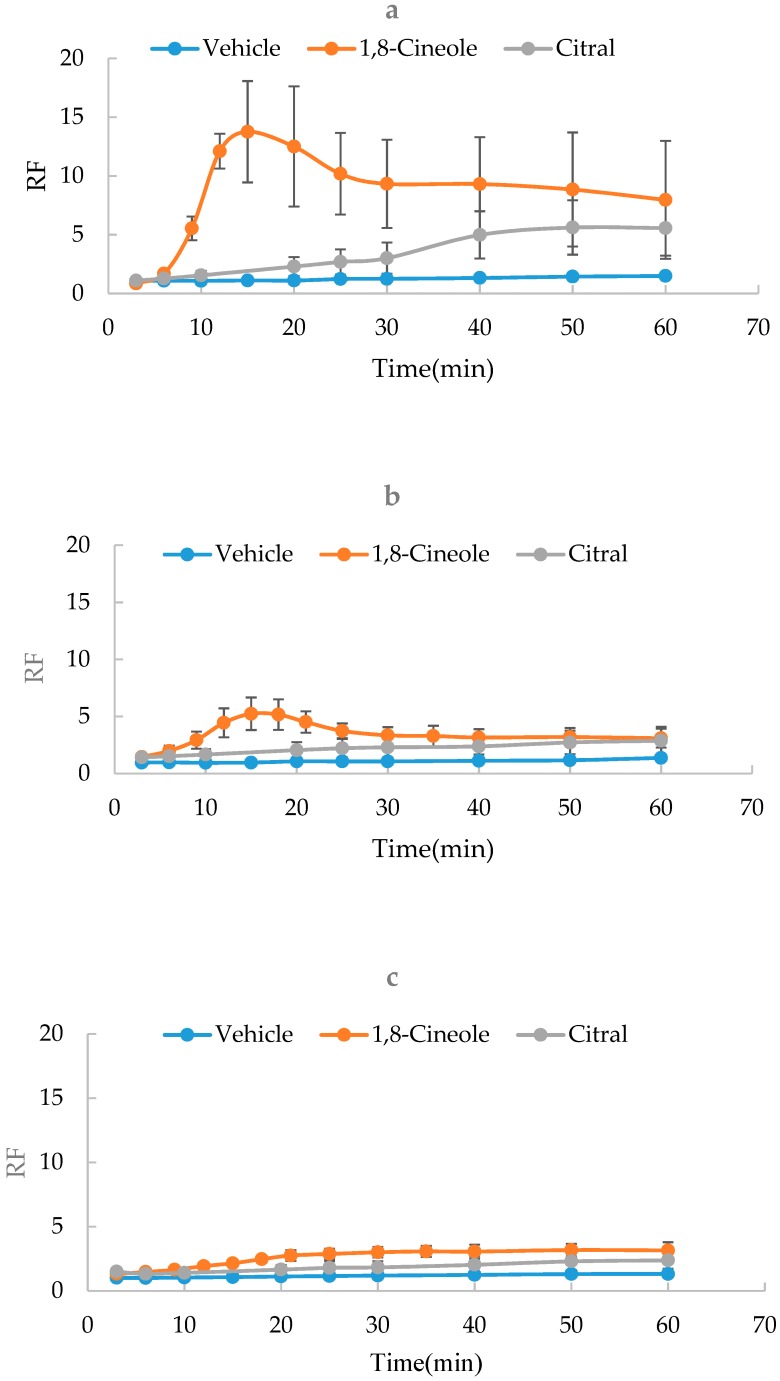
Effect of temperature on skin electrical resistance (SER) kinetics of 1,8-cineole, citral and vehicle (*n* = 3). (**a**) 37 °C; (**b**) 32 °C; (**c**) 25 °C.

It was reported that SER values were determined under 32–37 °C [17]. To evaluate the effect of temperature, the SER kinetics of 1,8-cineole (5%), citral (5%) and vehicle were measured at 25, 32 or 37 °C, respectively. As shown in Figure 1, results showed that there were the significant differences in the kinetics among three groups. The temperature of 37 °C was considered as the best one to perform SER kinetic experiments based on the highest rate of kinetics.

#### 2.2.2. Effect of the Concentration of PBS on SER Kinetic Measurement

**Figure 2:**
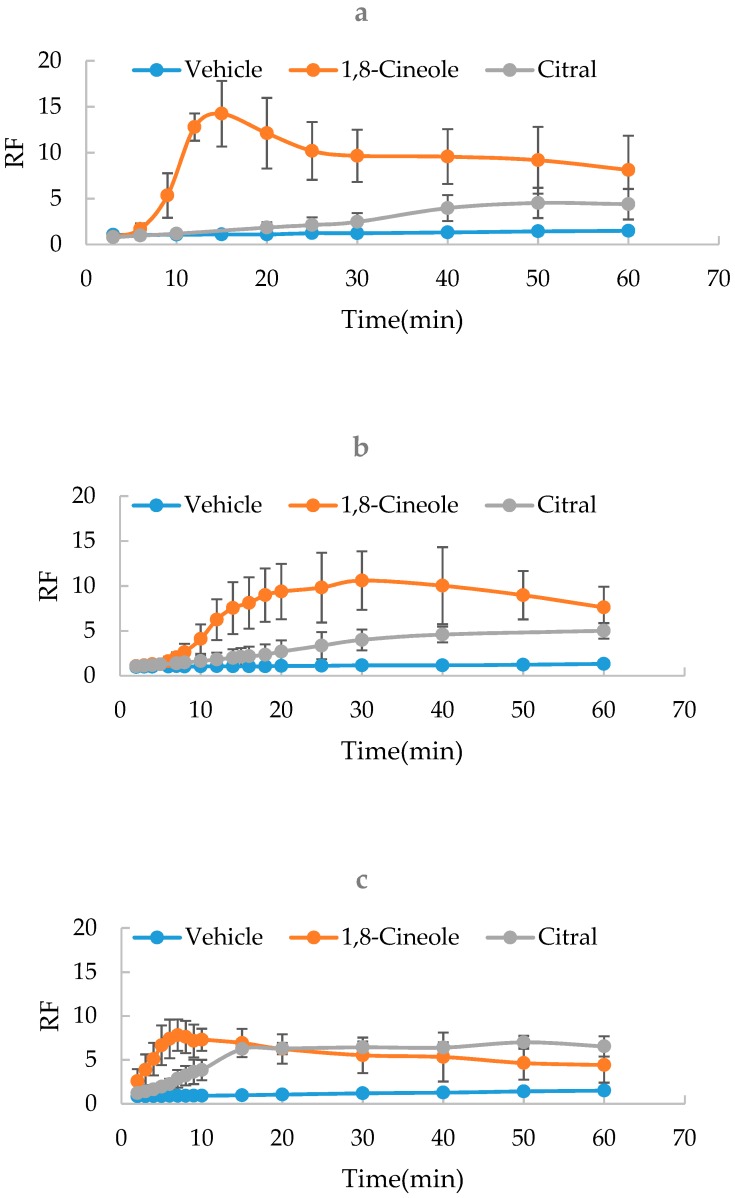
Effect of the phosphate buffered saline (PBS) concentration on SER kinetics of 1,8-cineole, citral and vehicle (*n* = 3). (**a**) PBS1; (**b**) PBS2; (**c**) PBS3.

Theoretically, the SER values are directly related with the concentration of phosphate buffered saline (PBS), the solvent of the donor and acceptor solution. The isotonic PBS1 was prepared and further diluted by 2 or 4-fold by water to obtain PBS2 and PBS3, respectively. Using an STY-1A Single-Sample Freezing Point Osmometer (TDTF Inc., Tianjin, China), the osmotic pressure values of PBS1, PBS2 and PBS3 were determined to be 297.67 ± 0.58, 150.10 ± 3.46, and 77.33 ± 6.03 mOsmol/kg H_2_O (*n* = 3), respectively. Using PBS of different concentrations (PBS1, PBS2 or PBS3) as the solvent, the SER kinetics of 1,8-cineole (5%), citral (5%) and vehicle were measured at 37 °C, respectively. As shown in Figure 2, it was found that the kinetics of SER were significantly modified by the three PBS with different concentrations. The increase of the PBS concentration seemed to result in the accelerated kinetic rate. Therefore, PBS1 was chosen as the preferred solvent to perform SER kinetic experiments.

#### 2.2.3. Effect of the Concentration of Terpenes on SER Kinetic Measurement

**Figure 3:**
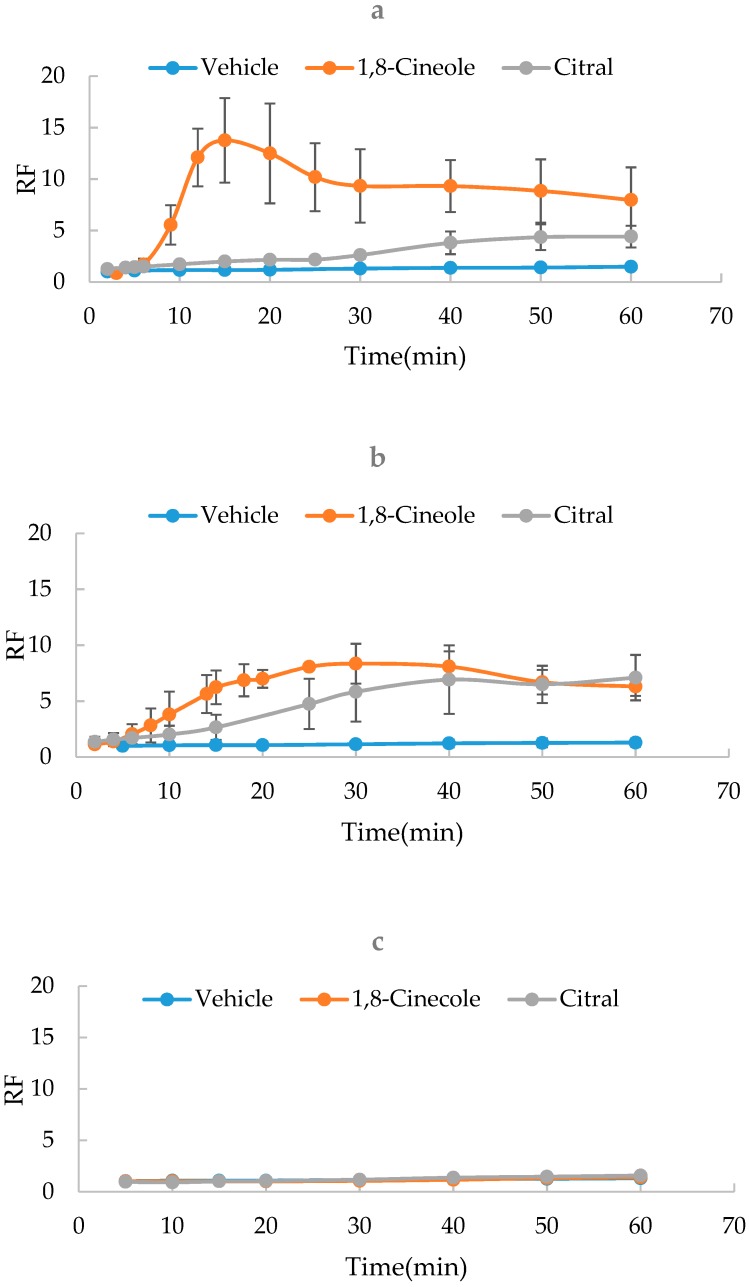
Effect of the terpene concentration on SER kinetics of 1,8-cinecole and citral (*n* = 3). (**a**) 5%; (**b**) 3%; (**c**) 1%.

To evaluate the modification of the concentrations of oxygen-containing terpenes on SER kinetics, 1,8-cineole and citral were measured at the concentrations of 1%, 3% and 5%, respectively. As shown in Figure 3, results indicated that the increased concentrations of 1,8-cineole or citral resulted in the more significant difference of SER kinetics. However, if the concentration was greater than 5%, it was difficult for the terpenes to dissolve in the vehicle. Therefore, the optimal terpene concentration was determined to be 5%.

### 2.3. Skin Electrical Resistance Kinetics of Oxygen-Containing Terpenes

**Figure 4:**
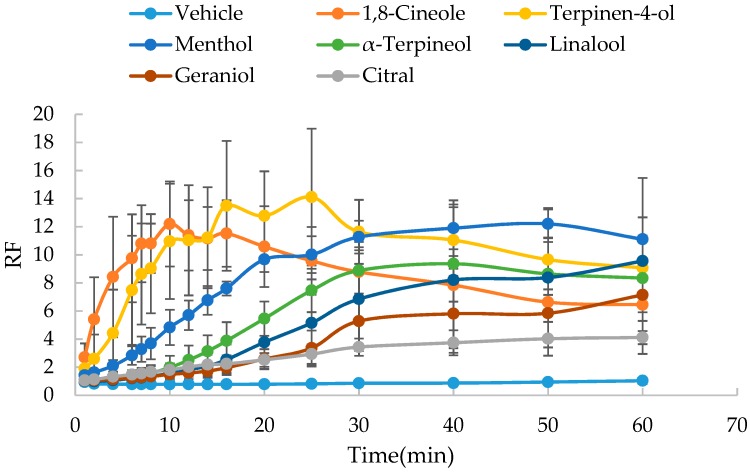
Effect of oxygen-containing terpenes dissolved in ethanol-PBS (1:1) on the resistance reduction factor (RF) values of excised rat skin (*n* = 5).

**Figure 5:**
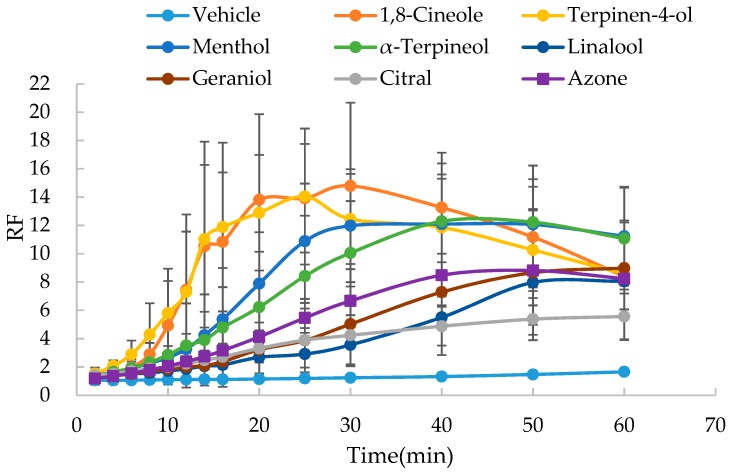
Effect of oxygen-containing terpenes dissolved in isopropanol-PBS (1:1) on the resistance reduction factor (RF) values of excised rat skin (*n* = 5).

As shown in Figure 4 and Figure 5, rapid increase in the the resistance reduction factor (RF) with time was observed with the presence of 5% oxygen-containing terpenes, while there was no significant change in RF in the vehicle. The slope values of the RF versus time curve are listed in Table 2 and Table 3. Azone at the concentration of 5% could not be dissolved in ethanol-PBS (1:1, *v*/*v*), which had been successfully applied as a vehicle for the SER measurement [12]. Therefore, azone was used as a positive control only in the vehicle of isopropanol-PBS (1:1, *v*/*v*).

Based on the evaluation of SER kinetics, all seven oxygen-containing terpenes were demonstrated to significantly (*p* < 0.05) reduce the skin barrier function and thus possess the permeation enhancement effect. Whether dissolved in ethanol-PBS (1:1) or isopropyl–PBS (1:1), based on the enhancement ratio (ER) values obtained by the comparison of the slope values of RF versus time curves, the reducing effect of cyclic terpenes (1,8-cineole, terpinen-4-ol, menthol and α-terpineol) on the skin barrier function was found to be greater than that of linear terpenes (linalool, geraniol and citral). However, compared to the use of isopropanol-PBS (1:1, *v*/*v*), the application of ethanol-PBS (1:1, *v*/*v*) resulted in more rapid reducing kinetics of SER. Accordingly, it was a little difficult for the accurate measurement of kinetic parameters of 1,8-cineole and terpinen-4-ol. Furthermore, to dissolve azone, the positive control, the vehicle of isopropanol-PBS (1:1, *v*/*v*) was used in the following studies.

In addition, for cyclic terpenes (1,8-cineole, terpinen-4-ol, menthol and α-terpineol), it was found that the RF values decreased obviously in the late phase of the curve, indicating the reversibility of their action on skin barrier function.

### 2.4. Molecular Simulation

In SC, a large number of ceramides are tightly arranged in the lipid bilayer due to the high degree of hydrogen bonding, resulting in the strong barrier function of SC. It is the hydrogen bond connection that forms the network at the head of ceramides. The tight network might be loosened by terpenes with a functional group that can denote or accept a hydrogen bond, promoting the skin permeation rate of drug [17]. Therefore, the oxygen-containing terpenes could disturb the ordered SC lipid organization by competitively forming hydrogen bonding with ceramides.

**Figure 6:**
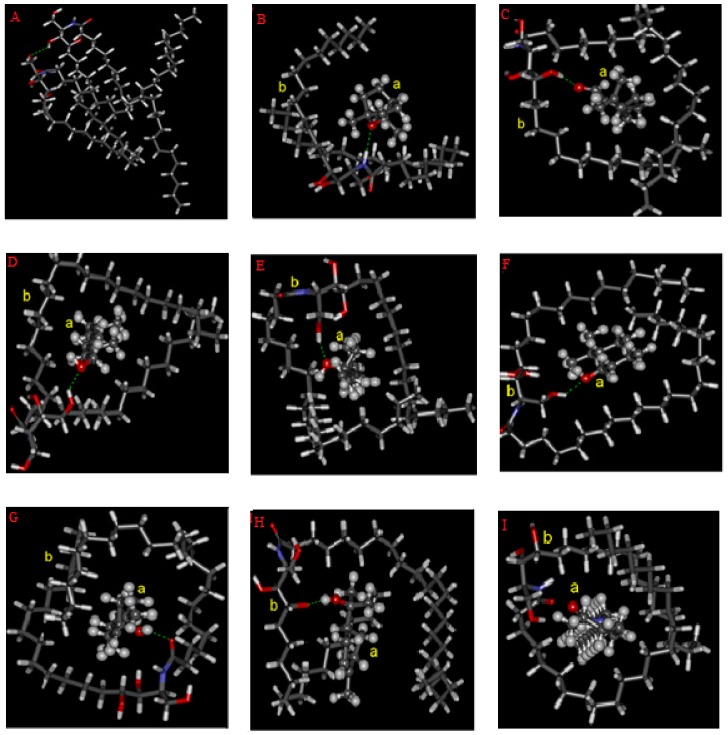
The stable complexes of ceramide 3 and oxygen-containing terpenes. (**A**) Ceramide 3 and Ceramide 3; (**B**) 1,8-Cineole, (**C**) Citral, (**D**) Geraniol, (**E**) Linalool, (**F**) Menthol, (**G**) Terpinen-4-ol, (**H**) α-Terpineol, (**I**) Azone. (Note: a. oxygen-containing terpenes; b. ceramide 3).

Ceramides are the major constituents in SC lipids and play a key role in maintaining the skin barrier function. Among them, ceramide 3 was often used to investigate the effect of PEs on the skin lipids due to its important role in the lipid organization of SC barrier [18,19,20]. Revealed by molecular simulation studies, all seven oxygen-containing terpenes could form stable hydrogen bonds with the head group of ceramide 3. The formed stable complexes are shown in Figure 6. Based on the optimal conformation, the calculated interaction energy values of the complexes are listed in Table 4. The lower the interaction energy, the more stable the complexes. The stability of the complex was approximately positively correlated with their penetration enhancement capacities evaluated by SER reducing kinetics.

**Figure 7:**
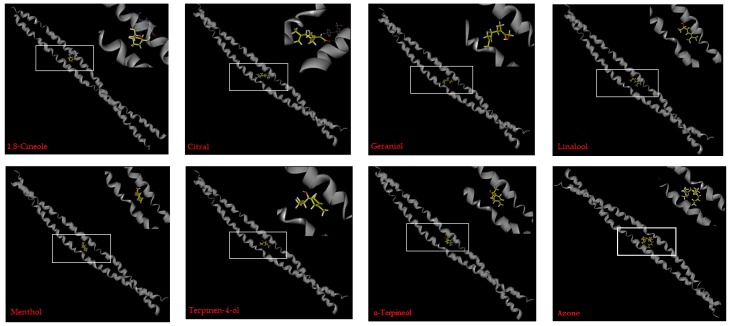
The stable complexes of oxygen-containing terpenes and keratin.

Molecular simulation was also employed to explore the possible interaction between oxygen-containing terpenes and skin protein. The PEs (seven terpenes and azone) were docked against the keratin and the minimum energy complexes are shown in Figure 7. The calculated docking energy is listed in Table 4. These terpenes interacted with keratin through van der Waals force and hydrophobic interaction, while azone presented a strong interaction with glutamine of keratin which was able to interfere with the interaction between water and keratin [21].

**Figure 8:**
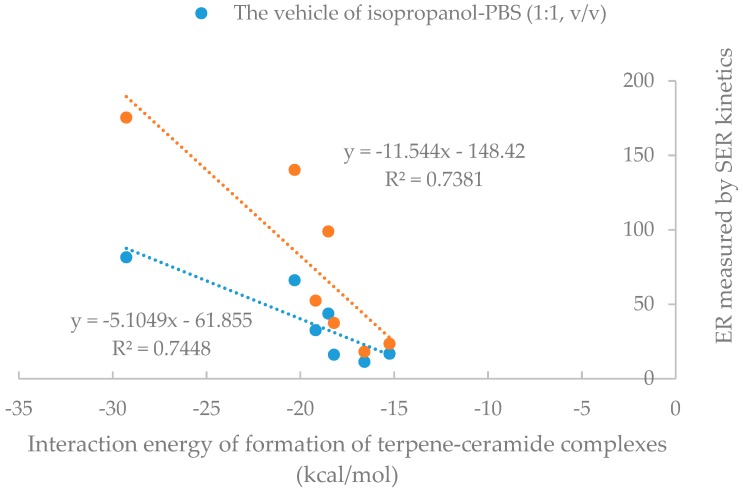
The correlation between enhancement ratio (ER) measured by SER kinetics and interaction energy of formation of terpene-ceramide complexes.

As shown in Table 4, the interaction between cyclic terpenes (1,8-cineole, terpinen-4-ol, menthol and α-terpineol) and ceramide was obviously stronger than that of linear terpenes (linalool, geraniol and citral). In summary, as shown in Figure 8, the results of SER measurement of oxygen-containing terpenes seemed to be directly correlated with their effects on SC lipids such as ceramides.

### 2.5. Attenuated Total Reflection-Fourier Transform Infrared Spectroscopy (ATR-FTIR) Studies

To elucidate the effect of oxygen-containing terpenes on the intercellular lipids in the SC, ATR-FTIR studies, which have already been proved to be a promising tool to study the spatial organization of SC lipids, were conducted [22,23]. ATR-FTIR stretching peaks near 2850 cm^−1^ (CH_2_ symmetric stretching vibration, Vs CH_2_) and 2918 cm^−1^ (CH_2_ asymmetric stretching vibration, Vas CH_2_) were measured after the application of different terpenes to the skin. Additionally, ATR-FTIR stretching peaks near 1650 cm^−1^ (Amide I) and 1547 cm^−1^ (Amide II), two strong keratin amide absorption peaks, were also determined. The changes of peak position are presented in Table 5.

The shift of a higher frequency occurs when the CH_2_ groups along the alkyl chain of lipids change from trans to gauche conformation, suggesting that the SC lipid is disturbed. In skin treated with cyclic terpenes, shifts were observed to CH_2_ symmetric and asymmetric stretching frequencies which were significantly higher than those observed with solvent alone (control), indicating fluidization of the lipid bilayer by cyclic terpenes. The higher the shifts, the higher the ratio of gauche to trans [24].

The sharp peaks at about 1650 and 1547 cm^−1^ corresponded to the absorption of the amide I and amide II of keratin, respectively. 1,8-Cineole seemed to have remarkable effects on both SC lipids and keratins.

### 2.6. Skin Cell Viability Assay

The epidermal keratinocyte HaCaT cells were usually employed to assess toxicity of PEs [25]. All examined oxygen-containing terpenes induced dose-dependent reductions in cellular viability. The half maximal inhibitory concentration (IC_50_) values, are shown in Table 6. The toxicity of azone, a well-established and standard chemical PE, was found to be prominently higher than the toxicities of all seven oxygen-containing terpene compounds, suggesting that the terpenes possessed relatively low skin irritation potential due to their natural origin. There was little difference between the toxicities of cyclic and linear terpenes. Furthermore, no correlation between penetration enhancement effect and skin cell toxicity was found. Among the seven terpenes, 1,8-cineole possessed the highest penetration enhancement effect and the lowest skin cell toxicity.

## 3. Discussion

The electrical resistance, namely electrical impedance, is a direct measure of skin permeability and can be applied to evaluate skin integrity [8]. It has been demonstrated by different research groups, and for different applications, the feasibility of using electrical resistance or conductivity as an efficient and effective indicator for screening of Pes [2,9,11,26]. The ordered structure and low permeability of SC lipids impart high electrical resistance to the SC, which was also proved in this study. Accordingly, the SER carries information that is of high relevance to the skin barrier function [2]. Dodecyl amino glucoside (DAG) has been proved to be a promising PE that acts through a reversible interaction with the SC lipids. Revealed by infrared studies, DAG acted on SC lipids and caused no change in protein conformation after 24 h treatment of SC. Following 2 h application of DAG, electrical resistance of human skin was found to be significantly decreased [10]. It can be concluded that the SER correlates well with skin barrier function, which is dependent on both the organization and the composition of SC lipids. Accordingly, skin barrier perturbation due to the penetration enhancement effect of PEs can be quantitatively analyzed by measuring SER kinetics. When the skin barrier is perturbed, the electrical resistance is expected to decrease. We further investigated the factors affecting the measurement of SER kinetics. According to this study, temperature, the concentration of PBS and the concentration of oxygen-containing terpenes can affect the SER kinetics. Whether in the vehicle of ethanol-PBS (1:1, *v*/*v*) [12] or isopropanol-PBS (1:1, *v*/*v*), the results of SER kinetics were found to be consistent. Finally, the optimal conditions for the SER measurement were obtained in this study for the first time.

Interestingly, based on SER kinetics, cyclic terpenes (1,8-cineole, terpinen-4-ol, menthol and α-terpineol) were demonstrated to possess higher penetration enhancement potential compared with linear terpenes (linalool, geraniol and citral). It is well known that the SC intercellular lipid domain is the site of action for many PEs, including oxygen-containing terpenes [27,28]. In the present study, the results of ATR-FTIR and molecular simulation also demonstrated that the main mechanism affecting SER measurement of oxygen-containing terpenes was mediated by disturbing SC lipid organization.

The boiling point of a terpene was found to be inversely related to its skin penetration enhancement effects. Terpenes with a low boiling point have relatively weaker intermolecular cohesive forces, suggesting that the oxygen of the functional group is mostly free, thus facilitating the competitive hydrogen bonding between the functional groups of terpenes and the SC ceramides [29]. Therefore, it was accepted that oxygen-containing terpenes with the highest boiling point values (citral and geraniol) possessed the lowest penetrationenhancement effect.

It should be noted that no obvious correlation was found between permeation enhancement effect and cytotoxicity (IC_50_) of skin cells of the seven oxygen-containing terpenes. 1,8-Cineole, possessing both high permeation enhancement efficacy and low cytotoxicity, was proved to be the best PE for TDD among the tested seven terpenes. Its permeation enhancement effect was more satisfactory compared with other PEs including polysorbate 80, azone, terpineol and N-methyl pyrrolidone [30].

## 4. Materials and Methods

### 4.1. Materials

Oxygen-containing terpenes, namely 1,8-cineole, citral, geraniol, linalool, menthol, terpinen-4-ol and α-terpineol (see Table 1 for a detailed information of the tested terpenes), all of purity >95.0%, were purchased from Aladdin Reagents Co., Ltd. (Shanghai, China). Azone was obtained from Sinopharm Chemical Reagent Co., Ltd. (Shanghai, China) and 3-(4,5-dimethylthiazol-2-yl)-2,5-diphenyltetrazolium bromide (MTT) was obtained from Sigma-Aldrich Inc. (St Louis, MO, USA). Deionized water was purified by a Direct-Q5 super purification system (Milipore, Billerica, MA, USA). Na_2_HPO_4_, NaH_2_PO_4_ and NaCl of analytical grade were purchased from Nanjing Chemical Reagent Corporation (Nanjing, China).

### 4.2. Animals

Sprague–Dawley rats (Male, 200 ± 20 g) were supplied by Shanghai Jiesijie Laboratory Animal Co. Ltd. (Shanghai, China) with the license number SCXK (Shanghai) 2013-0006. All of the procedures were performed in accordance with the Principles of Laboratory Animal Care and Use in Research (Ministry of Health, Beijing, China) and were approved by the Animals Ethics Committee of Nanjing University of Chinese Medicine (No. ACU1711102).

### 4.3. Skin Preparation

Skin preparation was carried out according to our previous study [31]. Prior to the experiment, the hair on abdominal area of the rats was shaved off with an animal hair clipper. After sacrificing the rats with excess ether inhalation, the full-thickness skin was then excised from the shaved zones. The adhering subcutaneous tissue was removed carefully and the prepared skin was subsequently washed with normal saline, wrapped in aluminum foil, and stored at −20 °C (used within two weeks).

### 4.4. Effect of SC on the Skin Electrical Resistance

To remove SC, the excised skin was immersed in 0.1% trypsin in PBS at room temperature for 10 h. Then, the SC sheets were carefully separated from remaining layers of skin. The skin as the control was treated with PBS at the same condition. To measure SER values, the treated rat skin was mounted on the bottom of the 24-well Transwell^TM^ culture insert (Corning Inc., New York, NY, USA) with the SC facing donor wells. The donor wells containing 150 μL PBS were then placed in the receiver plates containing 650 μL PBS. Following 1 h hydration by pH7.4 PBS at 37 °C, the values of the SER were directly measured by Millicell-ERS-2 Volt-ohm meter (Millipore, Billerica, MA, USA) connected to a pair of chopstick electrodes (STX01).

### 4.5. Effect of SC Lipids on the Skin Electrical Resistance

To investigate the effect of SC lipids, rat skin was mounted on the bottom of the 24-well Transwell^TM^ culture insert (Corning Inc., New York, NY, USA) with the SC facing donor wells. Then, 150 μL hexane:isopropanol (3:2) was added in the donor wells and stirred for 10 s, and this sequence was repeated 15 times to extract SC lipids. Subsequently, the extraction solvent was removed from the donor chamber completely and replenished with PBS. The donor wells containing 150 μL PBS were then placed in the receiver plates containing 650 μL PBS. The SER values were measured as described above.

### 4.6. Measurement of Skin Electrical Resistance Kinetics

To measure SER reduction kinetics, the excised rat skin was mounted on the bottom of the 24-well Transwell^TM^ culture insert (Corning Inc., New York, NY, USA) with the SC facing donor wells. The donor wells containing 150 μL PBS were then placed in the receiver plates containing 650 μL PBS. Following 1 h hydration by pH7.4 PBS at 37 °C, PBS in donor wells was replaced with 150 μL vehicle (ethanol-PBS (1:1, *v*/*v*) or isopropanol-PBS (1:1, *v*/*v*)) to measure the initial SER values (SER_0_). Then, the blank solution was removed and the test solution (5% terpene dissolved in vehicle) was added in the donor wells. Resistance measurements (SER_t_) were taken at different intervals in the range of 0–60 min. The resistance reduction factor (RF), which is defined as the ratio of the initial SER_0_ to the resistance values of the sample obtained at predetermined time (SER_t_), was calculated as given by: RF= SER_0_/ SER_t_ [12]. The RF values were plotted against time and the slope values of the linear part of the curve were calculated.

The factors affecting the measurement of SER kinetics of 5% 1,8-cineole, 5% citral and vehicle, including temperature, the concentration of PBS and the concentration of oxygen-containing terpenes, were investigated to obtain the optimum measurement conditions using ethanol-PBS (1:1, *v*/*v*) as vehicle [12]. To evaluate the effect of temperature, the SER kinetics were measured at 25, 32 or 37 °C, respectively. For the investigation of PBS concentration, the isotonic PBS1 (53 mM Na_2_HPO_4_, 13mM NaH_2_PO_4_ and 75 mM NaCl) was prepared and further diluted by 2 or 4-fold by water to obtain PBS2 and PBS3, respectively. Then, the SER kinetics were measured with different PBS. In addition, the SER kinetics were also measured at the terpene concentration of 1%, 3% and 5%, respectively.

Under the optimum measurement conditions, the SER kinetics of the seven oxygen-containing terpenes were measured and compared using ethanol-PBS (1:1, *v*/*v*) or isopropranol-PBS (1:1, *v*/*v*) as vehicle, respectively.

### 4.7. Molecular Simulation

The molecular interaction between terpenes and the SC lipids or proteins was carried out through molecular simulation [21].

Ceramide 3 and terpene molecules were first sketched using Chemdraw Ultra (Cambridge, MA, USA) and then the molecular simulations were performed on Discovery Studio 2.5 (DS 2.5, Accelrys, San Diego, CA, USA), which was used to generate the three-dimensional structures of these compounds. The ‘CDOCKER’ module of the DS 2.5 software package was used for semi-flexible docking using a CHARMm (Chemistry at HARvard Macromolecular Mechanics) force field and for molecular modeling. The process parameters of simulation were set in ‘‘Standard Dynamics Cascade’’, including minimization, minimization 2, heating, equilibration and production. The ten conformations obtained during docking were used for energy minimization calculations to determine the most probable conformation. The ceramide–enhancer complex could be formed after the above process, and the interaction energy values were generally negative, suggesting that the interaction of the ceramide 3 with terpene molecules was stable.

For the molecular docking between terpenes and the skin protein. The file for the 2B regions from keratin 5 and keratin 14 was obtained from the RCBS Protein Data Bank (PDB, 3TNU) [21]. Docking calculations were carried out in the CHARMm force field by using the Flexible Docking module of Discovery Studio 2.5 software (DS2.5). All torsions were allowed to rotate during docking. The LibDock was used to find the appropriate binding positions, orientations, and conformations of the ligands. Default parameters were used, except the maximum number of generate protein conformations was set at 2. The conformation method of generate ligand conformations was set at best and the max hits to save on docking was set at 3. The best docking type was chosen based on the binding energy scores. The corresponding heat of formation was calculated.

### 4.8. Attenuated Total Reflection-Fourier Transform Infrared Spectroscopy (ATR-FTIR) Studies

ATR-FTIR studies were performed in a similar manner to what was reported previously [24,31]. The excised skin which was cut into approximately 1 cm^2^ pieces soaked in the vehicle of isopropanol-PBS (1:1, *v*/*v*) with or without 5% various oxygen-containing terpenes at 32 °C for 24 h. The treated skin samples were washed with distilled water and then blotted dry. The infrared spectra of skin samples were obtained using Fourier transform infrared spectroscopy (FTIR-230 spectrometer, JASCO Co., Tokyo, Japan) equipped with an ATR unit (ATR-500/M, JASCO Co., Tokyo, Japan). The spectrum recorded under the following parameters: an average of 32 scans with a resolution of 2 cm^−1^ and the wavenumber range of 4000–650 cm^−1^.

### 4.9. Skin Cell Viability Assay

Human epidermal keratinocytes (HaCaT cell lines) were supplied from KeyGen Biotech Co. (Nanjing, China). MTT assay was used to evaluate the cellular toxicity of different oxygen-containing terpenes on epidermal keratinocytes. The well-established and standard PE azone was employed to compare and evaluate the cytotoxicity of these terpenes. The cells were incubated in complete culture medium which consists of Dulbecco’s Modified Eagle Medium (DMEM) with 10% fetal bovine serum and 100 U/mL penicillin/ streptomycin in a cell incubator at 37 °C and 5% CO_2_. HaCaT cells were seeded into 96-well plates at a density of 7000 cells per well. After 12 h, the cells were incubated with varying concentrations of terpenes in culture medium with 1% dimethyl sulfoxide (DMSO) at 37 °C for 24 h. Then, the medium was replaced with fresh medium containing 20 μL MTT solution (5 mg/mL in PBS). Following 4 h of incubation in CO_2_ incubator, the medium was eliminated and 150 μL DMSO was added to dissolve the formazan crystals. Then, the absorbance was read at 490 nm using a Chromate-4300 microplate spectrophotometer (Awareness Technology Inc., Palm City, FL, USA). Cells that did not receive any drug (control) were considered as having 100% cell growth and growth from treated cells was compared with this value (percent growth versus control).

### 4.10. Statistical Analysis

The results were expressed as mean ± S.D. Statistical comparisons were made using Student’s *t*-test and the chosen level of significance was *p* < 0.05.

## 5. Conclusions

Measurement of the changes in SER has been widely used as an efficient technique to screen PEs. The SER measurement was proved to be directly dependent on the SC barrier, especially SC lipids. Several factors, such as temperature, the concentration of PBS and the concentration of terpenes, were found to significantly affect the measurement of SER kinetics. The results of SER measurement of seven oxygen-containing terpenes, including four cyclic terpenes and three linear terpenes, showed that the effect on skin barrier function of cyclic terpenes was more significant than that of linear terpenes. Results of molecular simulation and ATR-FTIR analysis indicated that cyclic terpenes could act on SC lipids. The results of SER measurement of oxygen-containing terpenes seemed to be directly correlated with their effects on SC lipids such as ceramides. Moreover, among the seven oxygen-containing terpenes, 1,8-cineole which can affect both SC lipids and proteins has been demonstrated to possess both the highest penetration enhancement effect and lowest cell toxicity. In summary, the measurement of SER kinetics could be applied as an efficient method for the efficient evaluation of PEs mainly acting on SC lipids. Further research might be directed to the development of cyclic terpenes as PEs.

## Figures and Tables

**Table 1:** Physicochemical parameters of the tested oxygen-containing terpenes.

Terpene	Chemical Formula	MW	logP	Boiling Point (°C)	Chemical Structure
1,8-Cineole	C_10_H_18_O	154.249	2.82	174	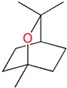
Citral	C_10_H_16_O	152.233	3.17	229.0	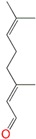
Geraniol	C_10_H_18_O	154.249	3.28	229.5	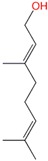
Linalool	C_10_H_18_O	154.249	3.28	198.5	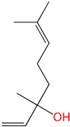
Menthol	C_10_H_20_O	156.265	3.2	215.4	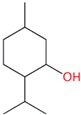
Terpinen-4-ol	C_10_H_18_O	154.249	2.99	209	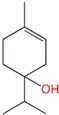
α-Terpineol	C_10_H_18_O	154.249	2.79	217.5	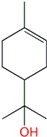

**Table 2:** The enhancement ratio (ER)values of different oxygen-containing terpenes calculated by skin electrical resistance (SER) kinetics with the use of the vehicle of ethanol- phosphate buffered saline (PBS) (1:1, *v*/*v*) (*n* = 5).

Penetration Enhancer (PE)	The Interval of the Linear Part of the Curve (min)	Slope of Resistance Reduction Factor (RF) Versus Time Curve (min^−1^)	ER = Slope_terpene_/Slope_vehicle_
Vehicle	0–30	0.0054 ± 0.0032	1.00
1,8-Cineole	0–10	0.9649 ± 0.2547 ***	175.35
Terpinen-4-ol	0–10	0.7571 ± 0.3113 ***	140.20
Menthol	0–20	0.5338 ± 0.1542 ***	98.85
α-Terpineol	0–30	0.2830 ± 0.0801 ***	52.41
Linalool	0–30	0.2018 ± 0.1106 **	37.37
Geraniol	0–25	0.1266 ± 0.0538 **	23.44
Citral	0–30	0.0976 ± 0.0441 ***	18.07
Azone	-	-	-

** *p* < 0.01, *** *p* < 0.001 vs the vehicle group. Note: the linear regression coefficient (r) values of the linear part are in the range of 0.9562–0.9980.

**Table 3:** The ER values of different oxygen-containing terpenes calculated by SER kinetics with the use of the vehicle of isopropanol-PBS (1:1, *v*/*v*) (*n* = 5).

PE	The Interval of the Linear Part of the Curve (min)	Slope of RF Versus Time Curve (min^−1^)	ER = Slope_terpene_/Slope_vehicle_
Vehicle	0–30	0.0096 ± 0.0010	1.00
1,8-Cineole	0–20	0.7825 ± 0.3946 ***	81.51
Terpinen-4-ol	0–25	0.6343 ± 0.2758 ***	66.07
Menthol	0–30	0.4189 ± 0.1607 ***	43.64
α-Terpineol	0–30	0.3117 ± 0.1077 ***	32.47
Linalool	0–30	0.1541 ± 0.0557 ***	16.05
Geraniol	0–30	0.1610 ± 0.0931 ***	16.77
Citral	0–30	0.1081 ± 0.0263 ***	11.26
Azone	0–30	0.2030 ± 0.0363 ***	21.15

*** *p* < 0.001 vs the vehicle group. Note: The linear regression coefficient (r) values of the linear part are in the range of 0.9673–0.9886.

**Table 4:** Interaction energy of formation of terpene-ceramide/keratin complexes.

Interaction Energy (kcal/mol)	Azone	Cyclic Terpenes				Linear Terpenes		
		1,8-Cineole	Menthol	Terpinen-4-ol	α-Terpineol	Citral	Geraniol	Linalool
Terpenes and ceramide ^a^	−22.16	−29.27	−18.50	−20.63	−19.18	−16.58	−15.24	−18.20
Terpenes and keratin	−22.43	−24.39	−15.33	−12.66	−14.80	−16.12	−20.39	−12.50

Note: ^a^ The interaction energy between ceramides was determined to be −13.28 kcal/mol.

**Table 5:** Peak positions in Attenuated Total Reflection-Fourier Transform Infrared Spectroscopy (ATR-FTIR) spectra of skin samples for the treatment of different oxygen-containing terpenes (*n* = 4).

Terpenes	Vas CH_2_	Vs CH_2_	Amide I	Amide II
Blank	2918.19 ± 0.00	2849.92 ± 0.20	1649.87 ± 0.12	1547.73 ± 0.15
Vehicle	2918.20 ± 0.34	2850.50 ± 0.21	1650.04 ± 0.67	1547.82 ± 0.48
1,8-Cineole	2919.65 ± 0.21 *	2851.17 ± 0.21 *	1649.08 ± 0.24 *	1546.85 ± 0.69
Citral	2917.88 ± 0.28	2850.52 ± 0.04	1651.01 ± 0.76	1547.13 ± 0.35
Geraniol	2918.04 ± 0.07	2850.20 ± 0.13	1649.08 ± 0.40	1547.02 ± 0.17 *
Linalool	2918.84 ± 0.46	2851.28 ± 0.13 *	1648.11 ± 0.71 *	1547.66 ± 0.60
Menthol	2920.13 ± 0.24 *	2850.85 ± 0.22	1650.04 ± 0.58	1547.87 ± 0.43
Terpinen-4-ol	2918.92 ± 0.21 *	2850.93 ± 0.25 *	1649.08 ± 0.68	1548.78 ± 0.57
α-Terpineol	2919.81 ± 0.56 *	2850.77 ± 0.30	1649.18 ± 0.67	1548.17 ± 0.32
Azone	2922.06 ± 0.68 *	2851.49 ± 0.41 *	1648.11 ± 0.32 *	1547.41 ± 0.67

* Represents the values significantly different from that of vehicle (*p* < 0.05).

**Table 6:** The half maximal inhibitory concentration (IC_50_) (μg/mL) values of oxygen-containing terpenes against HaCaT cells after 24 h incubation (*n* = 6).

Azone	Cyclic Terpenes				Linear Terpenes		
	1,8-Cineole	Menthol	Terpinen-4-ol	α-Terpineol	Citral	Geraniol	Linalool
7.55 ± 0.09	1701.97 ± 13.21	405.93 ± 3.47	732.60 ± 13.22	424.53 ± 4. 86	42.63 ± 2.10	172.01 ± 3.40	860.94 ± 4.08

## References

[1] Chen X. (2018). Current and future technological advances in transdermal gene delivery. Adv. Drug Deliv. Rev..

[2] Karande P., Jain A., Mitragotri S. (2006). Relationships between skin’s electrical impedance and permeability in the presence of chemical enhancers. J. Control. Release.

[3] Proksch E., Brandner J.M., Jensen J. (2008). The skin: An indispensable barrier. Exp. Dermatol..

[4] Lane M.E. (2013). Skin penetration enhancers. Int. J. Pharm..

[5] Chen J., Jiang Q.D., Chai Y.P., Zhang H., Peng P., Yang X.X. (2016). Natural terpenes as penetration enhancers for transdermal drug delivery. Molecules.

[6] Sapra B., Jain S., Tiwary A.K. (2008). Percutaneous permeation enhancement by terpenes: Mechanism view. AAPS J..

[7] Van Smeden J., Janssens M., Gooris G.S., Bouwstra J.A. (2014). The important role of stratum corneum lipids for the cutaneous barrier function. Biochim. Biophys. Acta..

[8] Davies D.J., Heylings J.R., McCarthy T.J., Correa C.M. (2015). Development of an in vitro model for studying the penetration of chemicals through compromised skin. Toxicol. Vitro.

[9] Karande P., Jain A., Mitragotri S. (2004). Discovery of transdermal penetration enhancers by high-throughput screening. Nat. Biotechnol..

[10] Kopečná M., Macháček M., Prchalová E., Štěpánek P., Drašar P., Kotora M., Vávrová K. (2017). Dodecyl amino glucoside enhances transdermal and topical drug delivery via reversible interaction with skin barrier lipids. Pharm. Res..

[11] Rachakonda V.K., Yerramsetty K.M., Madihally S.V., Robinson R.L., Gasem K.A.M. (2008). Screening of chemical penetration enhancers for transdermal drug delivery using electrical resistance of skin. Pharm. Res..

[12] Yerramsetty K.M., Rachakonda V.K., Neely B.J., Madihally S.V., Gasem K.A.M. (2010). Effect of different enhancers on the transdermal permeation of insulin analog. Int. J. Pharm..

[13] Wato K., Hara T., Yamana K., Nakao H., Inagi T., Terada K. (2012). An insight into the role of barrier related skin proteins. Int. J. Pharm..

[14] Southwell I.A., Russell M., Smith R.L., Brophy J.J., Day J. (2003). Melaleuca teretifolia chemovars: New Australian sources of citral and 1,8-cineole. J. Essent. Oil Res..

[15] Heard C.A., Kung D., Thomas C.P. (2006). Skin penetration enhancement of mefenamic acid by ethanol and 1,8-cineole can be explained by the ‘pull’ effect. Int. J. Pharm..

[16] Mutalik S., Parekh H.S., Davies N.M., Udupa N. (2009). A combined approach of chemical enhancers and sonophoresis for the transdermal delivery of tizanidine hydrochloride. Drug Deliv..

[17] Wang J., Dong C., Song Z., Zhang W., He X., Zhang R., Guo C., Zhang C., Li F., Wang C. (2016). Monocyclic monoterpenes as penetration enhancers of ligustrazine hydrochloride for dermal delivery. Pharm. Dev. Technol..

[18] Imura T., Sakai H., Yamauchi H., Kaise C., Kozawa K., Yokoyama S., Abe M. (2001). Preparation of liposomes containing Ceramide 3 and their membrane characteristics. Colloids Surf. B Biointerfaces..

[19] Rerek M.E., van Wyck D., Mendelsohn R., Moore D.J. (2005). FTIR spectroscopic studies of lipid dynamics in phytosphingosine ceramide models of the stratum corneum lipid matrix. Chem. Phys. Lipids.

[20] Chen Y., Wang J., Cun D., Wang M., Jiang J., Xi H., Cui H., Xu Y., Cheng M., Fang L. (2013). Effect of unsaturated menthol analogues on the in vitro penetration of 5-fluorouracil through rat skin. Int. J. Pharm..

[21] Liu X., Peng Q., Li S., Liu C., Zhao Y., Zhao Y., Fang L. (2017). Time dependence of the enhancement effect of chemical enhancers: Molecular mechanisms of enhancing kinetics. J. Control. Release.

[22] Laugel C., Yagoubi N., Baillet A. (2005). ATR-FTIR spectroscopy: A chemometric approach for studying the lipid organization of the stratum corneum. Chem. Phys. Lipids.

[23] Monti D., Tampucci S., Zucchetti E., Granchi C., Minutolo F., Piras A.M. (2018). Effect of Tumor Relevant Acidic Environment in the Interaction of a N-hydroxyindole-2-Carboxylic Derivative with the Phospholipid Bilayer. Pharm. Res..

[24] Furuishi T., Kato Y., Fukami T., Suzuki T., Endo T., Nagase H., Ueda H., Tomon K. (2013). Effect of terpenes on the skin permeation of lomerizine dihydrochloride. J. Pharm. Pharm. Sci..

[25] Welss T., Basketter D.A., Schröder K.R. (2004). In vitro skin irritation: Facts and future. State of the art view of mechanisms and models. Toxicol. Vitro.

[26] Karande P., Mitragotri S. (2002). High throughput screening of transdermal formulations. Pharm. Res..

[27] Ibrahim S.A., Li S.K. (2010). Chemical enhancer solubility in human stratum corneum lipids and enhancer mechanism of action and stratum corneum lipid domain. Int. J. Pharm..

[28] Chantasart D., Pongjanyakul T., Higuchi W.I., Li S.K. (2009). Effects of oxygen-containing terpenes as skin permeation enhancers on the lipoidal pathways of human epidermal membrane. J. Pharm. Sci..

[29] Zhou W., He S., Yang Y., Jian D., Chen X., Ding J. (2015). Formulation, characterization and clinical evaluation of propranolol hydrochloride gel for transdermal treatment of superficial infantile hemangioma. Drug Dev. Ind. Pharm..

[30] Ameen D., Michniak-Kohn B. (2017). Transdermal delivery of dimethyl fumarate for Alzheimer’s disease: Effect of penetration enhancers. Int. J. Pharm..

[31] Jiang Q., Wu Y., Zhang H., Liu P., Yao J., Yao P., Chen J., Duan J. (2017). Development of essential oils as skin permeation enhancers: Penetration enhancement effect and mechanism of action. Pharm. Biol..

